# Mediterranean Lifestyle Adherence Reflects Coherent Behavioural Patterns Based on the MEDLIFE Index

**DOI:** 10.3390/nu18050832

**Published:** 2026-03-04

**Authors:** Giorgio Bertolazzi, Salvatore Gagliardo, Francesco Saverio Ragusa, Nicola Veronese, Mario Barbagallo, Ligia J. Dominguez

**Affiliations:** 1 Department of Medicina and Surgery, Kore University of Enna, 94100 Enna, Italy; giorgio.bertolazzi@unikore.it; 2 Department of Orthopedics and Traumatology, Sant’Andrea University Hospital, Sapienza University of Rome, 00189 Rome, Italy; salvatore.gagliardo92@gmail.com; 3 Geriatric Unit, Department of Internal Medicine and Geriatrics, University of Palermo, 90100 Palermo, Italy; francescosaverioragusa@gmail.com (F.S.R.); mario.barbagallo@unipa.it (M.B.); 4 Faculty of Medicine, Saint Camillus International University of Health Sciences, 00131 Rome, Italy; nicola.veronese@unicamillus.org; 5 Department of Psychology, Educational Science and Human Movement, University of Palermo, 90133 Palermo, Italy

**Keywords:** Mediterranean diet, network analysis, diet, lifestyle, physical activity, MEDLIFE

## Abstract

Background/Objectives: The Mediterranean diet (MeDiet) is widely recognised as one of the healthiest dietary patterns, associated with reduced risk of chronic diseases and increased longevity. Beyond its nutritional components, the Mediterranean lifestyle encompasses a broader set of culturally rooted behaviours that may contribute to its health benefits. This study aimed to assess adherence to the Mediterranean diet and lifestyle using the MEDLIFE index and to explore how dietary and lifestyle behaviours cluster into coherent behavioural patterns. Methods: We conducted an observational study among undergraduate students in health and sports sciences and a comparison group of older adults, using an anonymous questionnaire based on the MEDLIFE index. Data were analysed using a pattern-based approach combining network analysis and score-based enrichment to characterise behavioural profiles associated with different levels of Mediterranean lifestyle adherence. Results: Network-based analyses revealed a high degree of internal coherence among dietary and lifestyle behaviours traditionally associated with the Mediterranean lifestyle. In particular, dietary restraint behaviours (e.g., limitation of sugar, salt, and snack consumption) systematically co-occurred with recommended Mediterranean food choices, indicating that positive intake and self-regulation are part of a unified behavioural framework. Score-based stratification confirmed these patterns at the individual level, with low adherence characterised by the absence of key Mediterranean components and unhealthy lifestyle habits, and high adherence reflecting an integrated profile combining healthy food choices, moderation, and lifestyle practices. Conclusions: Adherence to the Mediterranean diet reflects a holistic lifestyle strategy rather than a collection of isolated dietary behaviours. These findings support public health approaches that target coherent behavioural patterns, integrating diet, self-regulation, and lifestyle habits, rather than focusing exclusively on individual dietary components.

## 1. Introduction

Introduced in the late 1950s by Ancel Keys [1], the Mediterranean diet (MeDiet) describes the traditional dietary practices historically prevalent across the Mediterranean region [2]. This dietary pattern is widely recognised as one of the healthiest models of eating and is characterised by a high consumption of fresh or minimally processed foods, including daily intake of fruits, vegetables, whole grains and olive oil; regular consumption of legumes, nuts and seeds; moderate intake of fish; limited consumption of dairy products, eggs, sweets and red meat; water as the main beverage; and moderate wine consumption with meals, in accordance with cultural and social traditions [3].

A substantial body of evidence has demonstrated that adherence to the Mediterranean diet confers protective effects against major non-communicable diseases, including cardiovascular disease [4,5,6], cancer [7,8], and diabetes [9], and is associated with reduced all-cause mortality [10,11]. These numerous benefits linked to the Mediterranean diet have been demonstrated through various scoring systems that combine nutritional components only, without directly accounting for non-nutritional factors such as physical activity, social interaction, or sleep quality [4,5,6,7,8,9,10,11].

Despite these well-established benefits, adherence to the Mediterranean diet has markedly declined over recent decades, including in Mediterranean countries themselves [12]. Young adults appear particularly vulnerable to this decline, as socio-cultural changes, unhealthy behaviours, and lifestyle transitions often lead to poorer diet quality and reduced adherence to traditional dietary patterns [13]. Diet quality in this age group is shaped by a complex interplay of demographic characteristics, sedentary behaviours, social influences, and family context [14]. Eating outside the home and in social settings has been associated with changes in dietary patterns [15], while risky alcohol consumption represents an additional concern among young populations [16].

Beyond dietary components alone, the Mediterranean lifestyle encompasses a broader set of culturally rooted behaviours. In addition to a predominantly plant-based diet, this lifestyle includes adequate night time sleep, short daytime naps (“siestas”), regular physical activity, and strong social interactions [2]. Accordingly, increasing attention has shifted from the Mediterranean diet as a purely nutritional model toward the Mediterranean lifestyle as an integrated behavioural construct. The Mediterranean Lifestyle (MEDLIFE) index was developed to capture this multidimensional concept by evaluating adherence across three domains: food consumption, dietary habits, and lifestyle behaviours, including physical activity, rest, and social interaction [17]. Unlike indices focused exclusively on dietary intake, MEDLIFE enables the joint assessment of dietary and non-dietary behaviours, offering a more comprehensive representation of Mediterranean lifestyle adherence. Higher MEDLIFE scores have been associated with reduced risk of metabolic syndrome and mortality [18,19], as well as lower incidence of depression [20], frailty [21], cardiovascular disease [22], diabetes [23], and overall chronic disease burden, as estimated by biomarkers of inflammation and oxidative stress [24].

Adherence to the Mediterranean diet among university students has been explored in several studies [25,26,27,28,29,30,31,32], although investigations focusing specifically on students in medical and health-related disciplines remain limited [25,26,27]. Importantly, most previous studies have primarily assessed dietary components, with limited consideration of broader lifestyle behaviours. Moreover, the extent to which dietary and lifestyle components cohere into structured behavioural patterns has rarely been examined. This represents a relevant gap, particularly among medical, nursing, and sport science students, who will become future health professionals and may influence health-related behaviours within the wider community.

From a methodological perspective, MEDLIFE is typically analysed as a composite score, implicitly assuming coherence among its components. Network-based analytical approaches offer a data-driven framework to explore behavioural interdependencies, allowing the identification of clusters of co-occurring behaviours without imposing predefined groupings. In this context, network analysis provides added value over traditional scoring or clustering methods by explicitly modelling relationships among individual lifestyle components and identifying modular structures that reflect behavioural coherence.

In this context, there is a need to move beyond the assessment of isolated dietary factors and to adopt integrative approaches that consider how dietary and lifestyle behaviours co-occur within coherent patterns. Accordingly, the novelty of the present study lies in the application of statistically validated network and modular analyses to MEDLIFE components, to empirically examine behavioural coherence within the Mediterranean lifestyle. Therefore, this study aimed to assess adherence to the Mediterranean diet and lifestyle using the MEDLIFE index in undergraduate medical and sports science students and a sample of their older relatives, and to explore how dietary and lifestyle behaviours cluster into structured behavioural profiles associated with different levels of Mediterranean lifestyle adherence.

## 2. Methods

### 2.1. Study Design and Participants

The study population consisted of undergraduate students enrolled in medical, nursing, and sports sciences programmes at the Department of Medicine and Surgery of the Kore University of Enna (Italy). In addition, a comparison sample of older adults, including relatives of participating students, was included to broaden the age range of the sample and increase behavioural heterogeneity. The sole inclusion criterion was enrolment in one of the aforementioned academic programmes at the time of the study. The only exclusion criterion was refusal to participate. This study adopted an observational cross-sectional design and aimed to evaluate adherence to the Mediterranean diet and lifestyle among students in health-related disciplines. The older adult subgroup was recruited as a convenience comparison sample (primarily relatives of participating students). No a priori sample size calculation was performed for this subgroup, as it was not intended for age-stratified inferential analyses.

### 2.2. Ethical Principles

All participants received detailed information about the purpose of the study and the type of data collected on the home page of the online questionnaire, as a prerequisite for participation. They were informed that participation was entirely voluntary, that no geolocation data were collected, and that they had the right to decline participation or withdraw their consent at any time without any negative consequences. Participation required explicit consent through selection of an acceptance box on the electronic informed consent form. Participants were also informed that all collected data would be processed in compliance with Italian Law No. 219 of 22 December 2017, the General Data Protection Regulation (GDPR; Regulation EU 2016/679), the Declaration of Helsinki on Ethical Principles for Medical Research Involving Human Subjects, and the Code of Ethics of the Italian National Federation of the Orders of Physicians, Surgeons and Dentists. Participants’ anonymity was guaranteed, and data were processed exclusively in anonymous form for research purposes. The study protocol was approved by the Institutional Review Board of the Kore University of Enna (approval code: UKE-IRBPSY-09.22.01).

### 2.3. Data Collection

The questionnaire used for data collection was administered through a secure web-based platform (WordPress). A structured questionnaire including multiple-choice and open-ended items was used to collect information on dietary habits and lifestyle behaviours. Data collection was conducted between March 2023 and July 2025. The questionnaire was based on previously validated instruments, including the MEDLIFE index, and was reviewed for clarity before administration. Participants accessed the questionnaire independently using personal devices (computers, tablets, or smartphones). To ensure data quality, completion of all questionnaire items was required. Responses were collected automatically and exported in tabular format for subsequent statistical analysis. No directly identifying data was collected; as mentioned in the ethical considerations, participation was voluntary and anonymous.

### 2.4. Sociodemographic Data

The first section of the questionnaire collected participants’ sociodemographic information, including age, sex, education level, citizenship, ethnicity, socioeconomic status, smoking status, and self-reported health conditions (i.e., hypertension, coronary artery disease, diabetes, hypercholesterolemia, hypertriglyceridemia, cancer, or none). Self-reported body weight and height were also recorded. Body mass index (BMI) was calculated as weight in kilograms divided by height in metres squared (kg/m^2^) and categorised according to World Health Organization criteria as underweight (<18.50 kg/m^2^), normal weight (18.50–24.99 kg/m^2^), overweight (25.00–29.99 kg/m^2^) and obese (≥30.00 kg/m^2^).

### 2.5. Mediterranean Lifestyle-MEDLIFE–Index

Sotos-Prieto et al. [17] developed the MEDLIFE index, a validated measure grounded in the principles of the Mediterranean Diet Pyramid promoted by the Spanish Mediterranean Diet Foundation. Unlike other Mediterranean diet adherence scores, which focus mainly on food intake, the MEDLIFE index adopts a more comprehensive approach by incorporating multiple dimensions of the Mediterranean lifestyle. The index consists of 28 items organised into three domains: (1) food consumption (15 items), (2) other dietary practices, including coffee intake, sugar-sweetened beverage consumption, and sodium restriction (7 items), and (3) physical activity, rest, social behaviours, and communal dining (6 items). Each item is scored dichotomously (0 or 1), yielding a total MEDLIFE score ranging from 0, indicating low adherence, to 28, indicating high adherence to the Mediterranean lifestyle.

### 2.6. Statistical Analysis

*Variable coding*: Dietary and lifestyle variables derived from the MEDLIFE questionnaire were coded as binary indicators to allow a unified and interpretable analytical framework. Variable coding was tailored to the nutritional role of each food item with respect to the Mediterranean diet, according to four main categories:

1. Foods for which excessive consumption leads to deviation from the Mediterranean diet. For these items, a binary variable was created indicating whether consumption was within the recommended moderate range:
  $$x.moderate=\left\{\begin{array}{c} 1\;moderate\;assumption\\ 0\;abuse \end{array}\right.$$
2. Foods for which insufficient consumption leads to deviation from the Mediterranean diet. For these items, a binary variable indicated adequate intake:
  $$X.recommended=\left\{\begin{array}{c} 1\;\mathrm{correct}\;\mathrm{assumption}\\ 0\;under\;consumption \end{array}\right.$$
3. Foods for which intermediate consumption represents adherence to the Mediterranean diet. When the original variable had three ordered categories (low, moderate, high), it was recoded into three mutually exclusive binary variables, allowing each consumption level to be explicitly represented in the analyses:
  $$X.low=\left\{\begin{array}{c} 1\;\mathrm{low}\;\mathrm{assumption}\\ 0\;other \end{array}\right.;\;X.moderate=\left\{\begin{array}{c} 1\;\mathrm{moderate}\;\mathrm{assumption}\\ 0\;other \end{array}\right.;\;X.high=\left\{\begin{array}{c} 1\;\mathrm{abuse}\\ 0\;\mathrm{other} \end{array}\right.$$
4. Food limitation behaviors. For those items, a binary variable was created to capture active dietary restraint:
  $$X.limit=\left\{\begin{array}{c} 1\;\mathrm{limitation}\\ 0\;unrestricted\;consumption \end{array}\right.$$

All remaining variables not falling into the categories above were coded as simple presence/absence indicators (1/0). This harmonized binary representation allowed consistent comparison across dietary and lifestyle dimensions while preserving meaningful distinctions between moderation, adequacy, excess, and restraint.

*Mediterranean Lifestyle Index (MEDLIFE) score*: For each participant, an overall MEDLIFE score was calculated by summing the binary indicators reflecting adherence to Mediterranean diet and lifestyle recommendations. Higher scores indicated greater adherence to the Mediterranean lifestyle pattern, integrating dietary components, moderation and limitation behaviors, and lifestyle habits such as physical activity. The resulting score showed a broad distribution across the study population. Participants were subsequently ranked according to their total MEDLIFE score and classified into three groups based on score quartiles. For downstream analyses, attention was focused on the two extreme groups: a low-adherence group (MEDLIFE score lower than the first quartile) and a high-adherence group (MEDLIFE score higher than third quartile), representing individuals with clearly distinct levels of Mediterranean lifestyle adherence.

*Statistical Regression*: Linear regression analyses were performed to examine the association between the total Mediterranean lifestyle score and selected variables not directly included in its calculation. The selected variables not directly included in the MEDLIFE index calculation were age, sex, body mass index (BMI), and smoking status. This approach allowed the evaluation of the direction and strength of the relationships between Mediterranean lifestyle adherence and key demographic and behavioural factors.

*Statistically validated network of variables*: To explore the interdependencies among dietary and lifestyle variables, all possible pairwise associations were tested using Fisher’s exact test. Association *p*-values were adjusted for multiple testing using the Bonferroni method, and only statistically significant associations (adjusted *p* < 0.05) were retained. Significant associations were represented as a statistically validated network (SVN), in which nodes corresponded to variables and edges indicated significant associations. This approach, which has been previously applied to identify robust association structures in complex systems, allows statistically supported relationships to be retained while controlling for multiple testing and reducing the inclusion of spurious links [33,34,35]. Positive and negative associations were distinguished based on the sign of the log odds ratio and were visualised accordingly. Overall, this framework enabled the identification of coherent patterns of co-occurring behaviours while filtering out random or weak associations.

*Modular organization of dietary and lifestyle variables*: To identify groups of variables that tended to co-occur systematically, the SVN was analyzed using a community detection approach based on the Infomap algorithm [36], implemented through the *igraph* R package. This procedure allowed the identification of behaviorally meaningful modules, defined as sets of strongly interconnected variables representing coherent dietary or lifestyle patterns rather than isolated habits. The resulting modular structure provided a higher-level description of the network and facilitated interpretation of dietary and lifestyle behaviours in integrated, behaviourally relevant terms.

*Score-based enrichment analysis*: To characterize the behavioral profiles associated with different levels of Mediterranean lifestyle adherence, enrichment analyses were performed comparing the low- and high-adherence groups. For each variable, Fisher’s exact test was used to assess the over- and under-represented variables within the two groups. This analysis allowed the identification of dietary and lifestyle factors that most strongly distinguished individuals with high versus low adherence to the Mediterranean lifestyle.

*Respondent Clustering*: Participants were grouped according to similarities in their questionnaire responses using hierarchical clustering based on Jaccard distance. The resulting clusters represented distinct response profiles, characterised by variables that were positively or negatively associated with each cluster.

*Software*: All the statistical analyses were run through the R software (v.4.5.2). The network was graphically represented using the Cytoscape software (v.3.10.4).

## 3. Results

### 3.1. General Characteristics of Study Participants and Exploratory Analysis

The general characteristics of the overall study sample are presented in Table 1, while detailed age-stratified characteristics (<30 and ≥30 years) are reported in Supplementary Table S1. Younger participants showed a higher prevalence of smoking and higher socioeconomic status compared with older participants. Most individuals in both age groups did not report common chronic conditions, with a slightly higher proportion among younger participants. Older participants had a higher mean BMI, based on self-reported data, and a higher mean MEDLIFE index score compared with younger participants.

**Figure 1:**
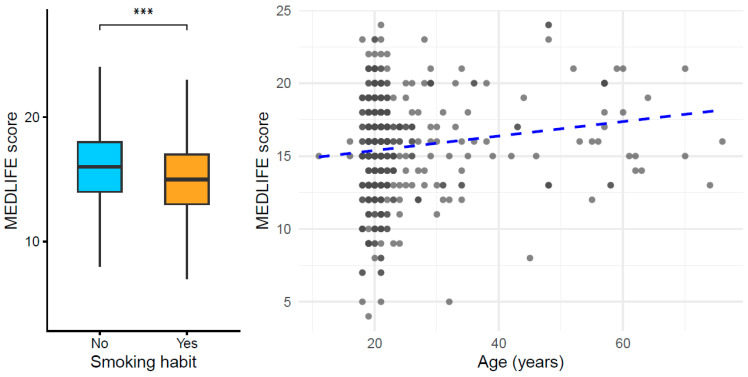
Association between MEDLIFE score, smoking status, and age. (***Left***) Distribution of MEDLIFE scores according to smoking habit (non-smokers vs. smokers). Boxplots represent the median and interquartile range, with whiskers indicating the distribution of values; the asterisks denote a statistically significant difference between groups (*p*-value = 0.00084). (***Right***) Scatterplot showing the relationship between age and MEDLIFE score. Each point represents an individual participant, and the dashed line indicates the fitted linear trend, highlighting the positive association between age and Mediterranean lifestyle adherence (*p*-value = 0.039).

Exploratory analyses focused on the relationship between selected individual characteristics and the overall MEDLIFE index score (Supplementary Table S2). Higher MEDLIFE scores were associated with a lower prevalence of smoking, as illustrated by the distribution of scores across smoking categories (Figure 1 *left panel*), and they showed a positive association with age (Figure 1 *right panel*).

### 3.2. Adherence to Mediterranean Diet and Lifestyle

#### 3.2.1. MEDLIFE Index Ranking

**Figure 2:**
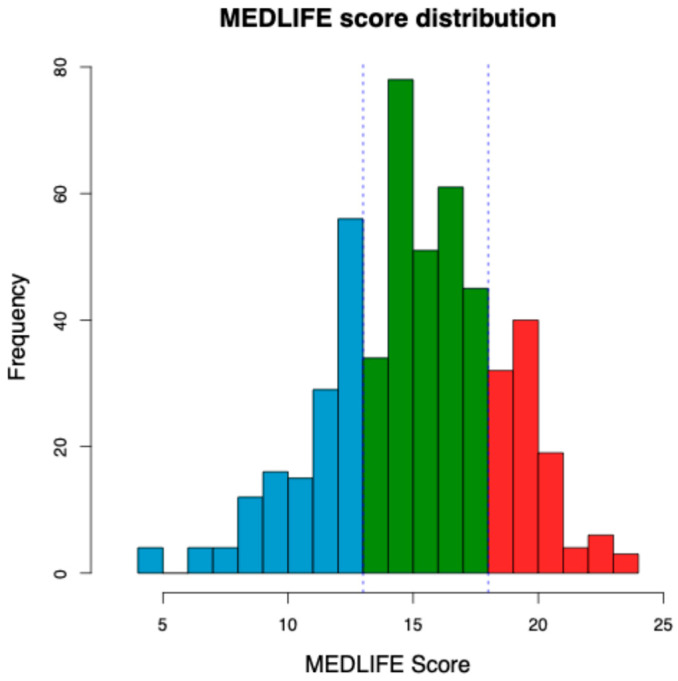
Distribution of the total MEDLIFE score in the study population. The histogram shows the distribution of the Mediterranean lifestyle adherence score, with participants classified according to score quartiles. Participants in the lowest quartile (low adherence to the Mediterranean diet) are highlighted in light blue (*n* = 84, 16.4%), whereas those in the highest quartile (high adherence) are highlighted in red (*n* = 104, 20.3%). Participants in the intermediate group are shown in green (*n* = 325, 63.3%).

Participants were ranked according to their total Mediterranean diet adherence score, which showed a broad distribution across the study population. Based on the score distribution, individuals were classified into three groups (Figure 2), and subsequent analyses focused on the two extreme categories: a low-adherence group and a high-adherence group, representing subjects with clearly distinct lifestyle profiles.

**Figure 3:**
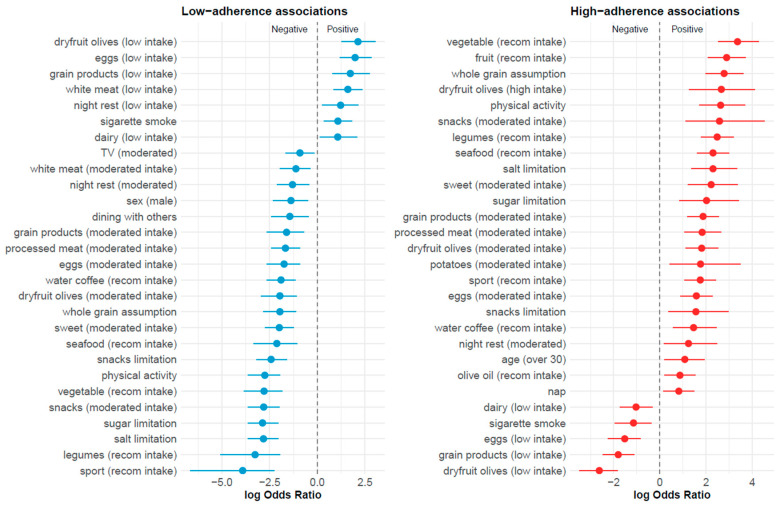
Forest plots showing variables significantly associated with low and high Mediterranean lifestyle adherence groups. The left panel presents associations with the low-adherence group, while the right panel presents associations with the high-adherence group. Points represent log odds ratios (logOR) and horizontal lines indicate 95% confidence intervals. Values to the right of zero (positive logOR) indicate variables over-represented in the corresponding adherence group, whereas values to the left of zero (negative logOR) indicate variables under-represented in that group. The vertical dashed line represents no association (logOR = 0). Only statistically significant associations after multiple testing correction are shown. *Abbreviations: recom.* = *recommended*.

Enrichment analysis showed that the low-adherence group was characterised by a systematic under-representation of behaviours typically associated with the Mediterranean diet, together with an over-representation of less favourable dietary and lifestyle patterns. Specifically, individuals in this group displayed markedly lower frequencies of vegetable, fruit, legume, whole-grain, and seafood consumption, as well as reduced engagement in physical activity and sport. Low intake of olives and dried fruit emerged as one of the strongest distinguishing features (Figure 3; Supplementary Table S3). In parallel, behaviours indicative of dietary imbalance and sedentary lifestyle were over-represented, including limited dietary restraint (e.g., lower prevalence of sugar and salt limitation), smoking, and prolonged television viewing. Overall, this group exhibited a coherent behavioural profile characterised by the absence of key Mediterranean dietary components combined with unfavourable lifestyle habits.

In contrast, the high-adherence group showed a highly consistent enrichment of behaviours aligned with the Mediterranean diet and a healthy lifestyle. This group was characterised by recommended consumption of vegetables, fruit, legumes, whole grains, and seafood, together with high intake of olives and dried fruit. Physical activity and sport participation were also markedly over-represented (Figure 3; Supplementary Table S3). Importantly, high adherence was not defined solely by the presence of healthy foods, but also by active dietary self-regulation, as individuals in this group were significantly more likely to limit sugar, salt, and snack consumption. Several unfavourable behaviours, including smoking, sedentary habits, and low-quality dietary patterns, were systematically under-represented. Taken together, these findings indicate that high Mediterranean diet adherence corresponds to an integrated behavioural profile combining dietary choices, moderation, and lifestyle practices, rather than isolated dietary components.

#### 3.2.2. Statistically Validated Network

**Figure 4:**
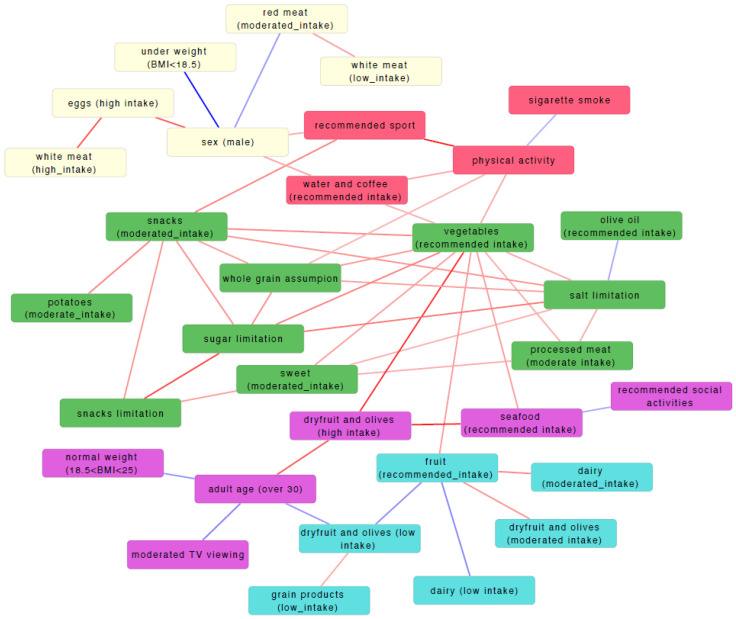
Statistically validated network (SVN) of dietary and lifestyle variables. Nodes represent individual variables, while edges indicate statistically significant associations between pairs of variables. Edge colour denotes the direction of the association, with red edges indicating positive associations and blue edges indicating negative associations. The node colors reflect the classification derived from the unsupervised clustering.

Using dichotomised MEDLIFE variables, we systematically screened all pairwise associations using Fisher’s exact tests and constructed a statistically validated network (SVN), in which edges represent significant positive or negative associations between variables (Figure 4; Table 2; Supplementary Table S4). The resulting network showed a high degree of internal coherence, with well-established healthy lifestyle behaviours organising into interconnected patterns. In particular, recommended vegetable consumption emerged as a central hub within the network, showing strong associations with whole-grain intake and multiple dietary restraint behaviours, consistent with a Mediterranean dietary pattern. In parallel, food limitation behaviours—such as limiting sugar, salt, and snack consumption—were tightly interconnected and also linked to healthier dietary choices, indicating that dietary restraint does not occur in isolation but rather as part of a coherent behavioural pattern. Overall, the presence of these expected and biologically plausible associations supports the robustness of the network structure and the internal consistency of the MEDLIFE-based behavioural framework.

#### 3.2.3. Modular Organization of Dietary and Lifestyle Variables

Analysis of the statistically validated network revealed that dietary and lifestyle variables organised into a limited number of behaviourally meaningful modules (Figure 4, Supplementary Table S5), indicating that habits tend to cluster into coherent patterns rather than occurring independently. Among the most relevant modules, Module 1 described a general healthy lifestyle profile, in which physical activity and sport participation clustered together with other positive behaviours and showed an opposite association with smoking. Module 2 grouped the limitations of unhealthy foods—such as sugar, salt, and snacks—with core components of the Mediterranean diet, including vegetables and whole grains, highlighting that dietary restraint and healthy food choices coexist within the same behavioural framework. Overall, these modules indicate that adherence to the Mediterranean diet is embedded within structured and interpretable behavioural constellations that integrate dietary choices, self-regulation practices, and lifestyle habits.

#### 3.2.4. Community Detection

**Figure 5:**
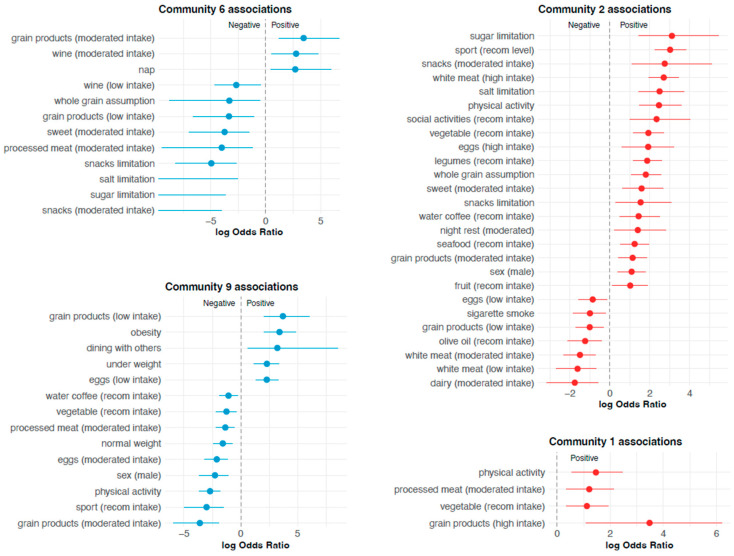
Forest plots of variables significantly associated with selected participant communities identified through hierarchical clustering. The panels display associations for communities 6 and 9 (characterised by lower Mediterranean lifestyle adherence) and communities 1 and 2 (characterised by higher Mediterranean lifestyle adherence). Points represent log odds ratios (logOR) and horizontal lines indicate 95% confidence intervals. Values to the right of zero (positive logOR) indicate variables over-represented within the corresponding community, whereas values to the left of zero (negative logOR) indicate variables under-represented in that community. The vertical dashed line represents no association (logOR = 0). Only statistically significant associations after multiple testing correction are shown. For some variables, the log odds ratio was not estimable (OR = 0) due to the complete absence of events in community 6; in these cases, only the upper bound of the 95% confidence interval is displayed. *Abbreviations: recom.* = *recommended*.

To further explore whether coherent behavioural profiles could also be identified at the individual level, an unsupervised clustering analysis was performed on participants’ responses (Supplementary Figure S1). Consistent with the score-based stratification, clusters associated with lower MEDLIFE scores (communities 6 and 9, Figure 5; Supplementary Table S6) were characterised by under-representation of key Mediterranean diet components and healthy lifestyle behaviours, including physical activity, sport participation, recommended intake of vegetables, legumes, whole grains, and dietary restraint practices. These clusters also showed over-representation of unfavourable behaviours such as smoking and sedentary habits. Conversely, clusters associated with higher MEDLIFE scores (communities 1 and 2, Figure 5; Supplementary Table S6) displayed a coherent enrichment of Mediterranean dietary components, active dietary self-regulation (e.g., sugar, salt, and snack limitations), physical activity, sport participation, and social activities. Overall, this analysis independently confirmed the presence of structured and contrasting behavioural profiles, in line with those identified through MEDLIFE score ranking and network-based analyses.

## 4. Discussion

The present study investigated adherence to the Mediterranean lifestyle in a population of health and sports science undergraduate students and a comparison sample of older adults, using the validated MEDLIFE index. This design allowed us to examine Mediterranean lifestyle adherence across a broader age range and behavioural spectrum. By integrating dietary and non-dietary lifestyle components within a single framework, this study provides a comprehensive assessment of Mediterranean lifestyle adherence that extends beyond isolated food-based indicators and single behavioural dimensions.

Several previous studies have examined adherence to the Mediterranean diet among university students [25,26,27,28,29,30,31,32], including a limited number focusing specifically on medical students [25,26,27]. Most of these investigations relied on dietary scores or nutrient-based indices and, in some cases, included selected adjustments for lifestyle behaviours such as physical activity, smoking, or alcohol consumption. However, none include other essential lifestyle factors like those assessed by the MEDLIFE index in our study, nor do they consider the combined impact of dietary and non-dietary factors. A former survey among 210 university students (105 Italian, 105 Spanish; mean age 27) reported nationality and gender-based differences in food consumption, with Italians eating more cereals and vegetables, and Spaniards consuming more fish and pulses. The study found a shift away from the traditional MeDiet, with being overweight linked to both physical activity and poor diet quality [28]. A study evaluated 284 Spanish students using the Mediterranean Diet Score (MDS), two 24 h food recalls, and a lifestyle questionnaire covering self-reported anthropometric data, housing type, smoking habits, and physical activity levels. The results showed that 72.5% had a normal BMI, and 75% were sedentary. The average MDS score was low (mean 4.0 ± 1.5), with key issues being low fruit/vegetable intake and high meat/dairy consumption. As a result, 96.1% had a ‘poor’ or ‘needs improvement’ diet, and only 5.3% had high adherence to the Mediterranean diet according to the MDS [29]. Another study evaluated 570 students aged 18–25 with the KIDMED test, showing that 9.5% had low, 62.1% intermediate, and 28.4% high adherence to the MeDiet. Students living at home had higher adherence (35.6%) than those in student residences (11.1%) or apartments (11.2%). Overweight students had lower adherence (15.5%) than normal weight students (8.5%) [30]. One more study assessed the link between MeDiet adherence, physical fitness, and body composition in 310 Spanish university students. The results showed that 24% had good MD adherence, linked to better cardiorespiratory fitness and muscle strength. Key adherence factors included >2 vegetables/day, using olive oil, <3 sweets/week, and ≥3 fruits/day. Students with good MeDiet adherence had higher protein intake and better physical fitness compared to those with lower adherence [31]. In a study aiming to assess early-risk alcohol consumption and dependence, MeDiet adherence, and emotional eating in 584 Spanish university students, 63.6% had low adherence to the MeDiet according to MEDAS, 26.2% were high-risk drinkers, 7.7% had alcohol dependence, and 38.6% were emotional eaters [32].

Regarding the few studies exploring adherence to the MeDiet in medical students, Fiore et al. evaluated 1038 medical students aged 18–34 years using a 16-item KIDMED questionnaire. The results show that 20.8% had ‘poor’, 56.5% ‘average’, and 22.7% ‘good’ adherence. Women had higher odds of better MeDiet adherence than men. The year in medical school had no significant effect [25]. Baydemir et al. conducted a cross-sectional study with 354 Turkish medical students assessing MeDiet adherence with the KIDMED index, along with data on BMI, residence, smoking, screen time, and exercise. They found a low average KIDMED score (3.8 ± 1.9), with no significant differences between first- and third-year students, concluding that medical students at Kocaeli University showed insufficient application of academic knowledge regarding healthy living [26]. In a cross-sectional study with anonymous surveys on demographics, lifestyle, and MeDiet adherence using the 14-point MEDAS among 589 Spanish medical students, González-Sosa et al. found that 58.9% had good MeDiet adherence, which was linked to age, higher academic years, alcohol consumption, and physical activity. They concluded that almost half of students had poor MeDiet adherence and that nutrition education is recommended [27].

While pattern-based and network-oriented approaches have been applied to the study of diet adherence and lifestyle-related behaviours [37,38], to the best of our knowledge, no previous studies have applied statistically validated network analysis to the MEDLIFE index to empirically examine interdependencies among its dietary and lifestyle components.

Beyond descriptive comparisons, the main contribution of this study lies in its analytical approach and in the interpretation of Mediterranean lifestyle adherence as a structured behavioural phenomenon. While the MEDLIFE index was conceptually designed to integrate dietary and lifestyle dimensions, the extent to which these components empirically cohere into structured behavioural patterns has not been formally tested. Network-based analyses revealed a high degree of internal coherence among dietary and lifestyle variables traditionally associated with healthy behaviours, thus providing empirical support for the internal consistency of the MEDLIFE framework. In particular, the central role of key Mediterranean dietary components within the network structure supports the presence of coherent behavioural configurations rather than isolated habits. Importantly, dietary restraint behaviours, including the limitation of sugar, salt, and snack consumption, did not emerge as marginal or compensatory practices, but were tightly interconnected with positive dietary choices. However, in the context of a young and health-educated population, these behaviours may reflect contemporary health-conscious norms or medicalised nutrition practices rather than exclusively traditional Mediterranean cultural habits, and should be interpreted accordingly.

The modular organisation of the variable network further reinforced this perspective. Behaviourally meaningful modules encompassed not only dietary components but also lifestyle dimensions, including physical activity, social engagement, and sedentary behaviours. In particular, modules linking dietary restraint with core Mediterranean foods highlighted the coexistence of self-regulation and positive food choices within the same behavioural framework. Moreover, the identification of a culturally rooted Mediterranean pattern—characterised by healthy food consumption, together with regular physical activity and an inverse association with cigarette smoking—suggests that Mediterranean lifestyle adherence reflects broader cultural and behavioural orientations rather than simple compliance with dietary recommendations.

Crucially, these network-level patterns were consistently mirrored at the individual level through score-based stratification and enrichment analyses. Individuals with low Mediterranean lifestyle adherence exhibited coherent profiles characterised by the absence of key Mediterranean foods, reduced physical activity, limited dietary restraint, and a higher prevalence of smoking and sedentary behaviours. Conversely, high adherence was associated with an integrated behavioural profile combining recommended food consumption, moderation, active dietary self-regulation, and healthier lifestyle practices. Notably, high adherence was not defined by extreme or restrictive behaviours, but rather by balance and moderation, which represent core principles of the Mediterranean lifestyle model.

### Strengths and Limitations

From a methodological standpoint, this study demonstrates the value of combining network-based representations of variable interdependencies with intuitive score-based stratification of individuals. Each analytical approach addressed a complementary objective: network analysis was used to characterise relationships among lifestyle components, while score-based analyses facilitated interpretation at the individual level. The convergence between network-derived modules and score-based enrichment profiles strengthens confidence in the robustness of the results and supports the interpretation of Mediterranean lifestyle adherence as a multidimensional behavioural pattern rather than a collection of independent dietary components. To the best of our knowledge, this is the first study to apply statistically validated network analysis to the MEDLIFE index to investigate behavioural interdependencies among dietary and lifestyle components in health and sports science university students.

However, several limitations should be acknowledged. The study was conducted as a single-centre, exploratory investigation, which limits the generalisability of the findings to other academic and cultural contexts. The subgroup of older adults was convenience-based and relatively small, and no a priori sample size estimation was performed for this subgroup; therefore, the study was not designed to support age-stratified or intergenerational inferential analyses. In addition, the cross-sectional design precludes causal inference, and the use of self-reported measures may introduce reporting bias. These factors require that the results be interpreted with appropriate caution. Future multi-centre studies with predefined sampling strategies and longitudinal designs are needed to confirm the reproducibility and temporal stability of the identified behavioural patterns. Accordingly, implications for public health should be interpreted with caution, while recognising the potential value of interventions targeting cohesive behavioural patterns rather than isolated lifestyle components.

## 5. Conclusions

This study shows that adherence to the Mediterranean diet and lifestyle emerges from coherent combinations of dietary and lifestyle behaviours rather than from isolated food choices. Specifically, network- and score-based analyses consistently identified integrated behavioural profiles characterised by the co-occurrence of recommended Mediterranean food consumption, dietary moderation and restraint, physical activity, and the absence of unhealthy behaviours such as smoking. Conversely, low adherence was associated with contrasting behavioural patterns combining unfavourable dietary choices and sedentary lifestyles.

These findings suggest that public health strategies aimed at promoting the Mediterranean lifestyle may benefit from targeting integrated behavioural patterns rather than focusing on single dietary components. In practical terms, interventions could combine dietary education with the promotion of physical activity and self-regulation skills, emphasising both positive food choices and the ability to limit unhealthy foods within everyday contexts. From a research perspective, future studies should investigate whether interventions addressing behavioural modules are more effective than single-behaviour approaches and should explore the stability and evolution of these behavioural patterns over time using longitudinal and network-based designs.

## Figures and Tables

**Table 1:** Main characteristics of the study participants. For numeric variables mean and standard deviation (±sd) were reported.

Parameter	Values
Sample size (*n*)	513
Mean age	24.3 ± 10.2
Mean BMI	22.9 ± 3.7
Smoking (%)	33.9
Females (%)	66.8
Mean MEDLIFE index	15.6 ± 3.4

**Table 2:** Summary statistics of the statistically validated network. A complete list of statistically significant pairwise associations is provided in Supplementary Table S4.

Network Feature	Description	Number (*n*)
Variables	Node included in the network	32
Positive associations	Positive links (red edges)	39
Negative associations	Negative links (blues edges)	11
Network modules	Identified variable clusters	5

## Data Availability

The data that support the findings of this study are available from the authors upon reasonable request.

## Supplementary Materials

The following supporting information can be downloaded at: https://www.mdpi.com/article/10.3390/nu18050832/s1, Figure S1: Dendrogram of participant clustering based on similarities in dietary and lifestyle responses; Table S1: Sociodemographic, clinical, and lifestyle characteristics of the study participants according to age group (<30 years and ≥30 years); Table S2: Results of the linear regression analysis examining the association between the MEDLIFE score and selected demographic and behavioural variables; Table S3: Variables enriching low- and high-adherence groups based on MEDLIFE score stratification; Table S4: Pairwise associations between dichotomous dietary and lifestyle variables included in the statistically validated network (SVN); Table S5: Within-module associations among dietary and lifestyle variables identified in the statistically validated network; Table S6: Variables enriching participant communities associated with low and high Mediterranean lifestyle adherence.

## Abbreviations

The following abbreviations are used in this manuscript:

BMI	Body mass index
MeDiet	Mediterranean diet
MEDLIFE	Mediterranean Lifestyle
SD	Standard deviation
SVN	Statistically Validated Network
