# Supplementary material for: Mediterranean Lifestyle Adherence Reflects Coherent Behavioural Patterns Based on the MEDLIFE Index
Source: Nutrients. 2026 Mar 4;18(5):832. doi: 10.3390/nu18050832 (PMC12986932; doi:10.3390/nu18050832)
Supplement: Supplementary file 1 [file nutrients-18-00832-s001.zip › nutrients-4118515-supplementary.pdf]

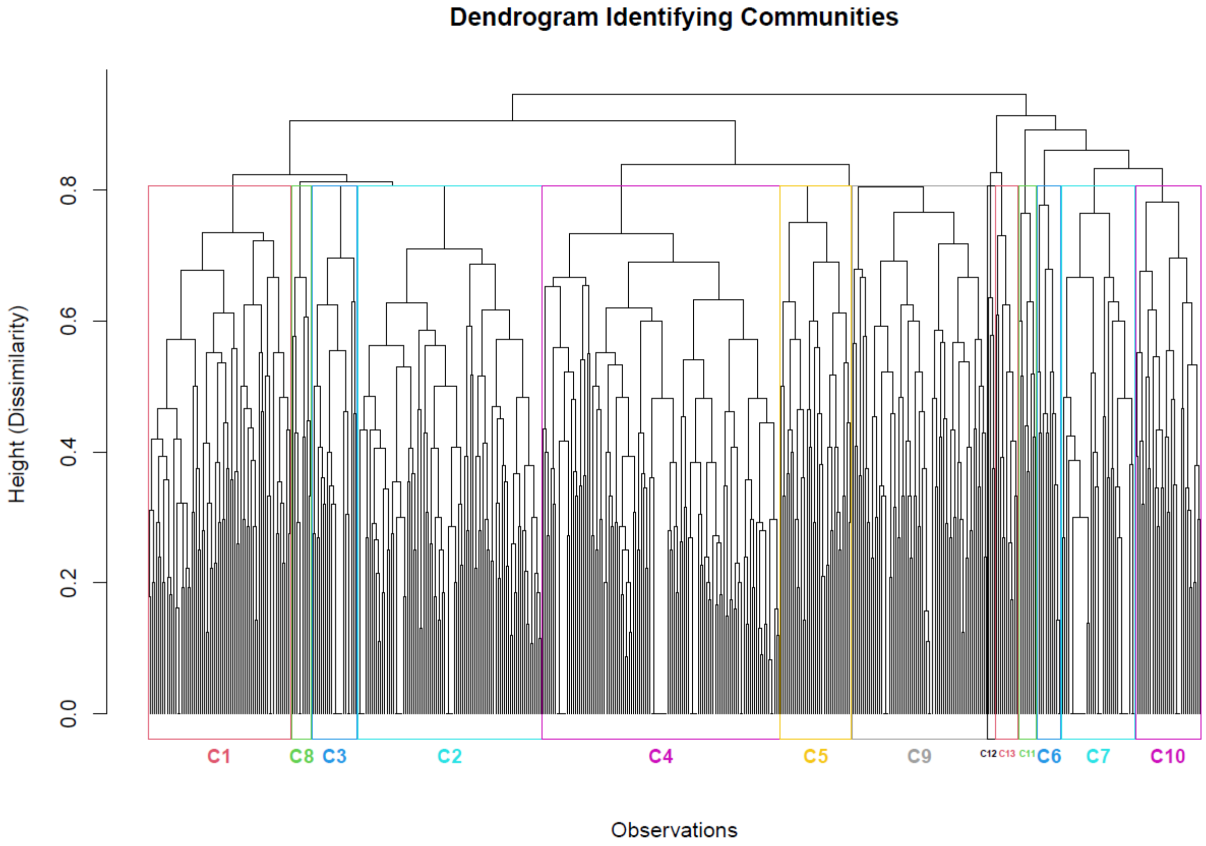

Figure S1: Dendrogram of participant clustering based on similarities in dietary and lifestyle responses.

The figure shows the hierarchical clustering of individual participants obtained using Jaccard distance applied to dichotomous questionnaire responses. The height of the branches represents the level of dissimilarity between observations. Colored boxes indicate the identified communities (C1–C13). These communities represent distinct response profiles characterised by different combinations of dietary and lifestyle behaviours.

Table S1. Sociodemographic, clinical, and lifestyle characteristics of the study participants according to age group (<30 years and ≥30 years).
This table reports a comparison between younger (<30 years) and older (≥30 years) participants. Values are expressed as percentages for categorical variables and as mean ± standard deviation (SD) for continuous variables. Characteristics include sociodemographic information, education level, socioeconomic status, smoking habit, self-reported health conditions, anthropometric measures, and MEDLIFE score.

Parameter	Age <30 years	Age >30 years
n	445	68
Mean age (SD)	20.9±2.3	46.9±12.8
Smoking (%)	36.4	17.6
Females (%)	67.9	60.3
Education level		
a. Elementary School (%)	0.67	0.0
b. High School (%)	92.13	38.23
c. University degree (%)	4.94	23.53
d. PhD or equivalent (%)	2.24	38.23
Socioeconomic status		
a. Poor or near-poor (%)	24.27	33.82
b. Lower-middle class (%)	34.38	44.11
c. Middle class (%)	20.44	8.82
d. Upper-middle class/rich (%)	20.89	13.23
Italian citizenship (%)	99.10	94.12
Hypertension (%)	0.0	10.29
Coronary artery disease (%)	2.24	1.47
Diabetes (%)	1.12	1.47
Cancer (%)	0.22	1.47
None (%)	96.17	88.23
Mean self-reported body weight in kg (SD)	63.68±11.97	72.07±13.70
Mean self-reported height in cm (SD)	167.97±9.44	168.85±8.91
Mean BMI (SD)	22.50±3.48	25.22±4.06
Mean MEDLIFE score (SD)	15.47±3.36	16.41±3.70

Abbreviations: BMI: body mass index; MEDLIFE: Mediterranean lifestyle index; SD: standard deviation

Table S2: Results of the linear regression analysis examining the association between the MEDLIFE score and selected demographic and behavioural variables. The table reports the results of simple linear regression models with the MEDLIFE score as the dependent variable and age, sex, smoking status, and body mass index (BMI) as independent variables. Coefficients (β), standard errors (SE), and p-values are shown. Statistical significance is indicated as follows: $p < 0.05$ (*), $p < 0.01$ (**), $p < 0.001$ (***).

variable	coeficient	SE	pvalue	significance level
(Intercept)	14,383	0,943	1,7E-43	
Age	0,039	0,015	0,012	*
Sex (male)	0,202	0,322	0,53	
Smoking	-1,077	0,321	0,00084	***
BMI	0,025	0,043	0,57	

Table S3. Variables enriching low- and high-adherence groups based on MEDLIFE score stratification. This table presents the results of the enrichment analysis comparing participants with low and high adherence to the Mediterranean lifestyle, as defined by the distribution of the MEDLIFE score. Variables are reported as positively or negatively associated with each adherence group, indicating over-representation (log OR>0) or under-representation (log OR<0) relative to the overall study population. Abbreviations: mod = moderated intake, rec = recommended intake

low-adherence enrichment					high-adherence enrichment				
**variable**	**OR**	**logOR**	**pval**	**adj.pval**	**variable**	**OR**	**logOR**	**pval**	**adj.pval**
salt.limit	0,14	-2,82	7,8E-15	4,7E-13	veg.rec	10,29	3,36	1,3E-20	8,2E-19
sugar.limit	0,14	-2,87	4,3E-14	1,3E-12	whole.grain	6,86	2,78	1,8E-15	5,5E-14
physical.activity	0,15	-2,75	7,3E-14	1,5E-12	legumes.rec	5,57	2,48	5,4E-14	1,1E-12
snacks.mod	0,14	-2,81	1,5E-12	2,3E-11	dryfruit.olives.low	0,16	-2,62	6,9E-14	1,1E-12
veg.rec	0,14	-2,79	2,3E-11	2,9E-10	fruit.rec	7,41	2,89	1,1E-13	1,3E-12
sport.rec	0,07	-3,91	4E-11	4,1E-10	seafood.rec	4,93	2,3	4,2E-12	4,2E-11
legumes.rec	0,1	-3,26	5,8E-10	5E-09	physical.activity	6,19	2,63	4,4E-11	3,8E-10
snacks.limit	0,19	-2,42	2,2E-09	1,7E-08	Community 2	4,76	2,25	1,3E-09	8,5E-09
Community 9	5,56	2,47	7,7E-09	5,2E-08	grain.products.mod	3,64	1,87	1,1E-08	6,6E-08
sweet.mod	0,25	-1,99	2,6E-08	1,5E-07	salt.limit	4,91	2,3	2,5E-08	1,4E-07
dryfruit.olives.low	4,38	2,13	3,9E-08	2E-07	grain.products.low	0,29	-1,8	3,3E-08	1,6E-07
eggs.low	3,94	1,98	9,9E-08	4,6E-07	dryfruit.olives.mod	3,51	1,81	3,4E-08	1,6E-07
water.coffee.rec	0,27	-1,9	1,5E-07	6,4E-07	sport.rec	3,37	1,75	1E-07	4,3E-07
whole.grain	0,26	-1,96	3,2E-07	1,3E-06	processed.meat.mod	3,56	1,83	3,8E-07	1,5E-06
dryfruit.olives.mod	0,26	-1,96	5,7E-07	2,2E-06	sweet.mod	4,67	2,22	4,1E-07	1,6E-06
fruit.rec	0	-Inf	7,8E-07	2,8E-06	eggs.mod	3	1,58	1,6E-06	5,6E-06
processed.meat.mod	0,31	-1,67	0,000003	9,8E-06	eggs.low	0,35	-1,52	5,4E-06	0,000018
eggs.mod	0,3	-1,74	0,0000043	0,000013	snacks.mod	5,97	2,58	0,000039	0,00012
seafood.rec	0,23	-2,12	0,0000072	0,000021	dryfruit.olives.high	6,32	2,66	0,000049	0,00015

Community 6	16,89	4,08	0,0000083	0,000023	sugar.limit	4,07	2,02	0,00009	0,00026
white.meat.low	3,04	1,6	0,0000095	0,000025	water.coffee.rec	2,76	1,46	0,00047	0,0013
grain.products.low	3,31	1,73	0,000048	0,00012	sigarette.smoke	0,45	-1,14	0,0024	0,0064
grain.products.mod	0,33	-1,61	0,00019	0,00047	Community 1	2,36	1,24	0,0035	0,0089
sex.M	0,38	-1,38	0,00093	0,0022	dairy.low	0,49	-1,03	0,0036	0,0089
night.rest.mod	0,41	-1,3	0,0019	0,0044	potatoes.mod	3,4	1,76	0,0049	0,011
dining.with.others	0,37	-1,44	0,0021	0,0046	snacks.limit	2,96	1,56	0,0057	0,012
sigarette.smoke	2,12	1,08	0,0024	0,005	olive.oil.rec	1,83	0,87	0,0082	0,016
white.meat.mod	0,46	-1,12	0,0025	0,0051	Community 7	2,73	1,45	0,0083	0,016
night.rest.low	2,32	1,22	0,0098	0,019	nap	1,77	0,82	0,011	0,021
TV.mod	0,53	-0,91	0,016	0,031	age.over30	2,11	1,08	0,014	0,026
dairy.low	2,09	1,07	0,02	0,037	night.rest.mod	2,37	1,24	0,017	0,031

Table S4: Pairwise associations between dichotomous dietary and lifestyle variables included in the statistically validated network (SVN).
This table lists all statistically significant associations between binary variables that constitute the links of the statistically validated network. Variables were coded as binary indicators reflecting adherence to the Mediterranean lifestyle (mod = moderated intake; rec = recommended intake; see Methods). Associations are reported as odds ratios (OR) with corresponding p-values. P-values were adjusted for multiple testing using the Bonferroni correction.
Abbreviations: mod = moderated intake, rec = recommended intake, OR = odds ratio

**Positive associations**					
**Variable 1**	**Variable 2**	**OR**	**logOR**	**pval**	**adj.pval**
physical.activity	sport.rec	17,91	4,16	2,9E-27	3,5E-24
snacks.limit	sugar.limit	9,74	3,28	2,2E-17	2,6E-14
salt.limit	sugar.limit	5	2,32	3,4E-12	4,1E-09
veg.rec	whole.grain	3,31	1,72	6,2E-11	0,000000074
veg.rec	sugar.limit	4,44	2,15	0,000000003	0,0000036
seafood.rec	veg.rec	3,11	1,64	9,2E-09	0,000011
salt.limit	whole.grain	3,03	1,6	0,000000015	0,000018
seafood.rec	dryfruit.olives.high	16,28	4,02	0,000000041	0,00005
whole.grain	sugar.limit	3,66	1,87	0,0000001	0,00012
red.meat.mod	white.meat.low	2,96	1,56	0,00000011	0,00013
veg.rec	snacks.mod	4,14	2,05	0,00000015	0,00018
sweet.mod	veg.rec	2,97	1,57	0,00000015	0,00019
dairy.mod	fruit.rec	4,14	2,05	0,00000021	0,00025
salt.limit	snacks.mod	3,59	1,84	0,00000021	0,00026
veg.rec	physical.activity	2,58	1,37	0,00000047	0,00057
eggs.high	white.meat.high	8,08	3,01	0,00000087	0,001
veg.rec	salt.limit	2,62	1,39	0,0000009	0,0011
processed.meat.mod	veg.rec	2,48	1,31	0,0000011	0,0013
dryfruit.olives.low	grain.products.low	2,51	1,33	0,0000012	0,0014

processed.meat.mod	salt.limit	2,53	1,34	0,0000012	0,0014
water.coffee.rec	physical.activity	2,69	1,43	0,0000012	0,0015
sport.rec	sex.M	2,61	1,38	0,0000016	0,0019
dryfruit.olives.mod	fruit.rec	3,45	1,79	0,0000022	0,0027
potatoes.mod	snacks.mod	4,19	2,07	0,0000023	0,0027
snacks.mod	sugar.limit	3,56	1,83	0,0000027	0,0033
sweet.mod	processed.meat.mod	2,54	1,34	0,0000029	0,0035
eggs.high	sex.M	7,46	2,9	0,0000034	0,0041
dryfruit.olives.high	veg.rec	13,33	3,74	0,0000069	0,0083
snacks.mod	sport.rec	4,31	2,11	0,0000071	0,0085
whole.grain	physical.activity	2,33	1,22	0,0000073	0,0088
fruit.rec	veg.rec	3,16	1,66	0,000009	0,011
water.coffee.rec	sex.M	2,81	1,49	0,000012	0,014
veg.rec	water.coffee.rec	2,47	1,31	0,000015	0,018
snacks.mod	snacks.limit	3,49	1,8	0,000017	0,02
sweet.mod	salt.limit	2,39	1,26	0,00002	0,023
sweet.mod	snacks.limit	2,97	1,57	0,00002	0,023
whole.grain	snacks.mod	3,03	1,6	0,000021	0,025
dryfruit.olives.high	age.over30	7,56	2,92	0,000022	0,026

negative associations					
**Variable 1**	**Variable 2**	**OR**	**logOR**	**pval**	**adj.pval**
red.meat.mod	sex.M	0,31	-1,68	1,5E-09	0,0000018
dairy.low	fruit.rec	0,24	-2,05	0,000000052	0,000062
TV.mod	age.over30	0,23	-2,14	0,000000052	0,000062
olive.oil.rec	salt.limit	0,37	-1,44	0,00000032	0,00039
dryfruit.olives.low	fruit.rec	0,27	-1,91	0,00000073	0,00088
sex.M	BMI.under.w.	0,05	-4,44	0,0000018	0,0022
age.over30	BMI.normal.w	0,29	-1,78	0,0000056	0,0067
dryfruit.olives.low	age.over30	0,3	-1,74	0,000013	0,015
physical.activity	sigarette.smoke	0,44	-1,2	0,000015	0,018
seafood.rec	social.activities.rec	0,38	-1,39	0,000035	0,042

Table S5: Within-module associations among dietary and lifestyle variables identified in the statistically validated network.
This table reports the significant associations between variables within each network module identified through community detection analysis. Associations are presented to describe the internal structure and coherence of the behavioural patterns characterising each module.
Abbreviations: mod = moderated intake, rec = recommended intake, OR = odds ratio

Module 1 associations							
X1	X2	OR	logOR	pval	adj.pval	association type	cluster
physical.activity	sport.rec	17,91	4,16	2,9E-27	3,5E-24	positive	1
water.coffee.rec	physical.activity	2,69	1,43	0,0000012	0,0015	positive	1
physical.activity	sigarette.smoke	0,44	-1,2	0,000015	0,018	negative	1

Module 2 associations							
X1	X2	OR	logOR	pval	adj.pval	association type	cluster
snacks.limit	sugar.limit	9,74	3,28	2,2E-17	2,6E-14	positive	2
salt.limit	sugar.limit	5	2,32	3,4E-12	4,1E-09	positive	2
veg.rec	whole.grain	3,31	1,72	6,2E-11	0,000000074	positive	2
veg.rec	sugar.limit	4,44	2,15	0,000000003	0,0000036	positive	2
salt.limit	whole.grain	3,03	1,6	0,000000015	0,000018	positive	2
whole.grain	sugar.limit	3,66	1,87	0,0000001	0,00012	positive	2
veg.rec	snacks.mod	4,14	2,05	0,00000015	0,00018	positive	2
sweet.mod	veg.rec	2,97	1,57	0,00000015	0,00019	positive	2
salt.limit	snacks.mod	3,59	1,84	0,00000021	0,00026	positive	2
olive.oil.rec	salt.limit	0,37	-1,44	0,00000032	0,00039	negative	2
veg.rec	salt.limit	2,62	1,39	0,0000009	0,0011	positive	2

processed.meat.mod	veg.rec	2,48	1,31	0,0000011	0,0013	positive	2
processed.meat.mod	salt.limit	2,53	1,34	0,0000012	0,0014	positive	2
potatoes.mod	snacks.mod	4,19	2,07	0,0000023	0,0027	positive	2
snacks.mod	sugar.limit	3,56	1,83	0,0000027	0,0033	positive	2
sweet.mod	processed.meat.mod	2,54	1,34	0,0000029	0,0035	positive	2
snacks.mod	snacks.limit	3,49	1,8	0,000017	0,02	positive	2
sweet.mod	salt.limit	2,39	1,26	0,00002	0,023	positive	2
sweet.mod	snacks.limit	2,97	1,57	0,00002	0,023	positive	2
whole.grain	snacks.mod	3,03	1,6	0,000021	0,025	positive	2

Module 3 associations

X1	X2	OR	logOR	pval	adj.pval	association type	cluster
red.meat.mod	sex.M	0,31	-1,68	1,5E-09	0,0000018	negative	3
red.meat.mod	white.meat.low	2,96	1,56	0,00000011	0,00013	positive	3
eggs.high	white.meat.high	8,08	3,01	0,00000087	0,001	positive	3
sex.M	BMI.under.w.	0,05	-4,44	0,0000018	0,0022	negative	3
eggs.high	sex.M	7,46	2,9	0,0000034	0,0041	positive	3

Module 4 associations

X1	X2	OR	logOR	pval	adj.pval	association type	cluster
seafood.rec	dryfruit.olives.high	16,28	4,02	0,000000041	0,00005	positive	4
TV.mod	age.over30	0,23	-2,14	0,000000052	0,000062	negative	4
age.over30	BMI.normal.w	0,29	-1,78	0,0000056	0,0067	negative	4
dryfruit.olives.high	age.over30	7,56	2,92	0,000022	0,026	positive	4
seafood.rec	social.activities.rec	0,38	-1,39	0,000035	0,042	negative	4

Module 5 associations

X1	X2	OR	logOR	pval	adj.pval	association type	cluster
dairy.low	fruit.rec	0,24	-2,05	0,000000052	0,000062	negative	5
dairy.mod	fruit.rec	4,14	2,05	0,00000021	0,00025	positive	5

dryfruit.olives.low	fruit.rec	0,27	-1,91	0,00000073	0,00088	negative	5
dryfruit.olives.low	grain.products.low	2,51	1,33	0,0000012	0,0014	positive	5
dryfruit.olives.mod	fruit.rec	3,45	1,79	0,0000022	0,0027	positive	5

Table S6: Variables enriching participant communities associated with low and high Mediterranean lifestyle adherence.
This table reports the enrichment analysis of variables characterising selected participant communities identified through clustering analysis. Communities 6 and 9 are associated with behavioural profiles diverging from the Mediterranean lifestyle, whereas communities 1 and 2 are characterised by behaviours consistent with Mediterranean lifestyle adherence. Variables are reported as positively or negatively associated with each community.
Abbreviations: mod = moderated intake, rec = recommended intake, OR = odds ratio

Low MEDLIFE score Communities

Community 6

variable	OR	logOR	pval	adj.pval
snacks.mod	0	-Inf	1,6E-10	7,9E-09
sugar.limit	0	-Inf	1,7E-09	0,000000043
snacks.limit	0,03	-4,95	0,00000023	0,0000038
salt.limit	0	-Inf	0,0000016	0,000019
sweet.mod	0,07	-3,74	0,0001	0,001
grain.products.mod	10,9	3,45	0,00038	0,0031
processed.meat.mo	0,06	-4	0,00045	0,0032
grain.products.low	0,1	-3,34	0,00058	0,0036
whole.grain	0,1	-3,31	0,0071	0,037
nap	6,47	2,69	0,0073	0,037
wine.mod	6,81	2,77	0,0083	0,038
wine.low	0,16	-2,68	0,01	0,042
legumes.rec	0	-Inf	0,011	0,043

Community 9

variable	OR	logOR	pval	adj.pval
physical.activity	0,15	-2,7	2,7E-11	1,4E-09
grain.products.low	13,1	3,71	2,6E-09	0,000000065
grain.products.mod	0,08	-3,6	1,1E-08	0,00000018
eggs.low	4,89	2,29	0,00000007	0,00000087
BMI.obese	10,6	3,41	0,00000036	0,0000036
sport.rec	0,12	-3,01	0,00000052	0,0000044
eggs.mod	0,23	-2,11	0,0000021	0,000015
sex.M	0,21	-2,27	0,00001	0,000064
BMI.under.w.	4,93	2,3	0,00003	0,00017
BMI.normal.w	0,34	-1,58	0,000069	0,00035
processed.meat.mo	0,39	-1,36	0,00047	0,0021
veg.rec	0,42	-1,26	0,0022	0,0091
dining.with.others	9,29	3,22	0,0052	0,02
water.coffee.rec	0,47	-1,08	0,0071	0,025

High MEDLIFE score Communities

Community 2

variable	OR	logOR	pval	adj.pval
sport.rec	8,12	3,02	1,2E-17	5,9E-16
white.meat.high	6,52	2,7	2,3E-14	5,9E-13
physical.activity	5,51	2,46	4,8E-09	0,000000079
salt.limit	5,63	2,49	0,000000032	0,0000004
veg.rec	3,8	1,93	0,000000045	0,00000045
legumes.rec	3,65	1,87	0,000000056	0,00000047
whole.grain	3,48	1,8	0,00000037	0,0000026
sugar.limit	8,61	3,11	0,0000029	0,000018
white.meat.mod	0,35	-1,5	0,000058	0,00029
snacks.mod	6,75	2,75	0,000062	0,00029
social.activities.rec	5,05	2,34	0,000064	0,00029
white.meat.low	0,33	-1,62	0,00029	0,0011
sweet.mod	3,01	1,59	0,00029	0,0011
seafood.rec	2,37	1,24	0,00037	0,0013
grain.products.mod	2,2	1,14	0,0012	0,0037
dairy.mod	0,3	-1,76	0,0012	0,0037
water.coffee.rec	2,72	1,44	0,0015	0,0042
olive.oil.rec	0,42	-1,24	0,0015	0,0042
sex.M	2,13	1,09	0,0019	0,005
eggs.high	3,77	1,92	0,0021	0,0053
grain.products.low	0,5	-1,01	0,0033	0,0078
snacks.limit	2,92	1,54	0,0098	0,022
sigarette.smoke	0,5	-1	0,01	0,022
eggs.low	0,55	-0,86	0,014	0,03
night.rest.mod	2,63	1,4	0,018	0,035
fruit.rec	2,03	1,02	0,021	0,04

Community 1

variable	OR	logOR	pval	adj.pval
dryfruit.olives.mod	Inf	Inf	1,6E-29	8E-28
dryfruit.olives.low	0	-Inf	4,3E-26	1,1E-24
physical.activity	2,76	1,46	0,00083	0,014
grain.products.high	11,19	3,48	0,0017	0,021
veg.rec	2,18	1,12	0,0029	0,029
processed.meat.mod	2,31	1,21	0,0039	0,032
